# John Billings' "Life"; by Dr. F. H. Garrison

**Published:** 1915-06-19

**Authors:** 


### A FATHER OF MODERN HOSPITALS.

### John Billings' "Life," by Dr. F. H. Garrison.

Under the title of "A Great Hospital Authority" we published in The Hospital of March 22, 1913, some account of the work of Colonel John Shaw Billings, whose death had occurred eleven days before. Army surgeon, hospital constructor, civil administrator, hygienist, and perhaps the most famous bibliographer which medical literature has ever had, all who know a great man when death reveals him (for death is usually more fortunate than life in this respect) will turn with eagerness to the "Memoir,"* which Putnam has now published, by Dr. Fielding H. Garrison. A "Memoir" is to English readers hardly suggestive of the scope of this capable book, in which all the public life and work of Dr. John Shaw Billings is straightforwardly told, from the early days when, aghast at the incomplete education which fortune was bequeathing to him, he began to teach himself Latin, till the crowning public labour of his life was undertaken when he accepted, as its first holder and organiser, the post of Director of the New York Public Library. The stupendous task of setting this great foundation on its feet by the amalgamation of three existing libraries, the organisation and labour required for the reproduction of a complete new catalogue for the entire collection, kept him busy till his death, and he died in harness in 1913.

In view of the comprehensive sketch of his public work which we published at the time of Dr. Billings' death, in the issue already referred to, we need not repeat the story here, though the activities which were crowded into it might tempt one, and will certainly tempt American readers, into exploring side issues of many kinds. For those who realise the importance of Dr. Billings' life, at least to hospital administrators, and who will therefore feel a grave defect in any institutional library which possesses no copy, one word of warning should be uttered. The immense chapter, no doubt inviting to Americans, entitled "Experiences of a Medical Officer during the Civil War," will hardly repay careful reading. The reason is that to Englishmen it offers but quotations from Dr. Billings' diary, jottings of the day, orders for marching, and so on, without any reference whatever to the war or the campaign—all of which is taken for granted. No doubt Americans are as familiar with their Civil War as we are with the campaign of Waterloo, but to Englishmen it is a closed book, which they must be coaxed to open. The result is that, owing to no background having been provided for Dr. Billings' diary (and not even a map is given), the diary form becomes extremely irritating, since there seems no excuse for so much detail with regard to a campaign of which no account is given, and to which only the baldest and scantiest references are made. All this is not to say that (granted a full knowledge of the movements of the two armies, and an interest in the success or failure of each) Dr. Billings' diary does not make the daily progress of events from the point of view of a busy officer of interest. There are many shrewd remarks which, particularly just now, are well worth reading. For instance, of his experience at Chancellorsville he writes: "It is one thing to provide for wounded when the troops are advancing and leaving the hospital behind, and quite another thing to fall back with your wounded when the troops are retreating." Again, he makes the following confession: "I like fighting tolerably well, although it is not half so exciting as I had supposed it would be. . . . After all, a big battle is a humbug—it takes 10,000 bullets to kill a man." What would he say to-day? Of life on campaign he says: "Of mental enjoyment there is little or none—altogether the life is beastly to the last degree—yet by no means unpleasant to a man who derives his pleasure from physical sources." One thing, however, comes very clearly out of his war experiences, and that is the very high ability Billings showed in these early days as an organiser; no situation was too difficult, no accommodation too scanty, no numbers of incoming wounded were so many but that he would create order out of chaos and get his staff at work in a few hours. We cannot leave this long chapter, however, without giving the author this excuse for, so to speak, taking the war and his readers' knowledge of the campaign for granted. Billings himself seemed to ignore the larger aspects of both entirely. He had his job, a tough one, which he performed with singular ability, and there the matter ended for him. Thus on page 133 it is hardly a surprise to read that "one of Billings' old army friends once said that never, in all the course of a long and intimate association, did he reveal his reasons for going into the war or discuss its issues after the struggle was over." Yet it gave to Billings the difficulties—in other words, the opportunity and training—which he was to develop to such good purpose later on.

So much had Billings' abilities impressed the authorities that in 1864 he was assigned to the Washington branch of the office of the Medical Director of the Army of the Potomac (with which he had been on active service). Here he was set to work to analyse its field reports. In short, he was gravitating towards the centre, and the next step in his career occurred in 1870, when he was placed in charge of the collection of books known as the Library of the Surgeon-General's Office, at which point his work in medical bibliography began. His work in public hygiene also started about this time, when his inspection of the Marine Hospital Service led to the publication of voluminous reports on "Barracks and Hospitals" and "The Hygiene of the United States Army." At this time, like most thinkers before the general

* *John Shaw Billings*: A Memoir by Fielding H. Garrison, M.D. Illustrated. G. P. Putnam and Sons, New York and London. 1915. Price $2.50 (10s.) net.

acceptance of Lister's and Semmelweiss' teaching, Billings believed that the life of a hospital building should be limited to twelve years.

His public position had become virtually assured when, in 1875, his plans for the construction of the Johns Hopkins Hospital, Baltimore, were selected by the trustees of that foundation, and the next twenty-five years of his life were spent in its construction and organisation. The chapter of Dr. Garrison's book in which this great work is described is of the utmost value as showing that Billings possessed the necessary combination which alone can make a man a great administrator—namely, a firm grasp of first principles to which all ideas must be referred; and, secondly, a practical experience of detail in organisation, construction, and design. As an example of the first we may quote the following sentence from his first report to the trustees of the Johns Hopkins Hospital on the plans which the architect had based on Billings' own requirements. "The hospital," he wrote, "should contribute to charity, to education, and science. First to charity. . . . Patients are to have the benefit of the best medical and surgical skill . . . and they are not to be subjected to any annoyances or depressing influences by being made a show of in any way." Thus Billings was perfectly clear in his own mind as to the mutual relation of treatment of the sick, medical education, and science, or, as we now call it, research. He placed them in the order of their importance, and organised his hospital and its medical school so as to carry out and not infringe this order. Again, his grasp of first principles is shown in the remark that "our buildings and machinery are simply tools . . . the moving and animating soul of the institution which is to do its work and determine its character consists of the brains to be put in it. . . . It depends upon not more than half-a-dozen men and one or two women." On the other hand, he clearly indicated the important effect of plan and arrangement on the staff and working of any institution. As an instance of his practical wisdom take his dictum on the medical school (at the time he wrote, remember, a new thing to be created on different lines from all the existing schools of the United States). "We can more certainly secure" patient investigators and skilled research workers "by showing them that they shall have space and apparatus to work with . . . and that any discoveries they make shall be properly published than by doubling their pay." Into his fascinating forecast of what a medical school should do in training *original* investigators, in supplying education for public sanitary officers, and into his then startling recommendations for branches dealing with mental disease and jurisprudence and pediatrics, we have not space to enter. It is very well related by Dr. Garrison, who describes the visits paid by Billings to Europe, largely from letters which have been preserved. These tours are illuminating, if only for the way in which they show how Billings always provided himself with a host who could help him to gather the particular information he was in search of, and thus made sight-seeing and hospitality only the atmosphere in which much hard work was being done. His suggestions on ventilation, and a multitude of other points, are illustrated in these letters, which again and again illuminate the whole voluntary system by such reminders as that a hospital should be not only an index of the civilisation and public spirit of the district which it serves, but also "an active force in the community." It must teach, he points out, many valuable lessons such as those of a standard of cleanliness and comfort for the sick, on the one hand, and no less the value of personal service to the healthy and the importance of a reverence for the dead and regard for their relatives on the other. Many such points are conveniently summarised in Dr. Billings' own "Description" of the Johns Hopkins Hospital, which is the father of a host of text-books on construction and ventilation.

The development of the medical school, which was in active working by 1893, or of the training school for nurses, opened in 1889, sufficiently indicated the large views of medical education and the position of medicine in America, which his great work as a medical bibliographer did so much to establish on a firm foundation. His work on the Surgeon-General's Library and Catalogue, heralded, in its completed form, by the issue of his "Specimen Fasciculus" in 1876, was thus an accompaniment of his already heroic labours towards the establishment of the Johns Hopkins Hospital. The appearance in 1880 of the first volume of the *Index Catalogue* marked an epoch in the development of medical literature, especially in America, since it gave to all medical writers, for the first time in the States, their materials in a comprehensive but accessible form. The technical knowledge, industry, and skilled arrangement necessary for the success of such a work marked Billings when the time came, in 1896, for the difficult task of "creating" the New York Public Library through the amalgamation of the Astor, Lennox, and Tilden foundations. As its director he became responsible for negotiating, planning, building, stocking, and cataloguing the new library, a vast work which cannot be gone into here, but enough in itself for one man's lifetime.

In this account of Dr. Garrison's life of Dr. Billings we have aimed rather at a presentation of the field of work covered than at a critique of the memoir as a biography, or an examination exclusively devoted to Billings' achievements as a hospital authority. Indeed, in the case of a typical man of action like Dr. Billings, the methods of Boswell, or even Lockhart, would hardly be suitable. Therefore, we think Dr. Garrison has done well to confine himself to a straightforward and conveniently arranged story of the public work of Dr. Billings, and to rely on the importance of that work to give value to his book and life to his picture. It is the sort of "life," we believe, that Dr. Billings would have approved, for he would have preferred to have its pages confined, as they are entirely, to the public work he performed as a pioneer in so many varied fields.

